# Molecular characterization of *KU70* and *KU80* homologues and exploitation of a *KU70*-deficient mutant for improving gene deletion frequency in *Rhodosporidium toruloides*

**DOI:** 10.1186/1471-2180-14-50

**Published:** 2014-02-27

**Authors:** Chong Mei John Koh, Yanbin Liu, Moehninsi, Minge Du, Lianghui Ji

**Affiliations:** 1 Biomaterials and Biocatalysts Group, Temasek Life Sciences Laboratory, 1 Research Link, National University of Singapore, Singapore 117604, Singapore

**Keywords:** Nonhomologous end-joining (NHEJ), *Rhodosporidium toruloides*, Oleaginous yeast, *Agrobacterium tumefaciens*-mediated transformation (ATMT), Homologous recombination

## Abstract

**Background:**

*Rhodosporidium toruloides* is a β-carotenoid accumulating, oleaginous yeast that has great biotechnological potential. The lack of reliable and efficient genetic manipulation tools have been a major hurdle blocking its adoption as a biotechnology platform.

**Results:**

We report for the first time the development of a highly efficient targeted gene deletion method in *R. toruloides* ATCC 10657 via *Agrobacterium tumefaciens*-mediated transformation. To further improve targeting frequency, the *KU70* and *KU80* homologs in *R. toruloides* were isolated and characterized in detail. A *KU70*-deficient mutant (∆ku70e) generated with the hygromycin selection cassette removed by the Cre-*loxP* recombination system showed a dramatically improved targeted gene deletion frequency, with over 90% of the transformants being true knockouts when homology sequence length of at least 1 kb was used. Successful gene targeting could be made with homologous flanking sequences as short as 100 bp in the ∆ku70e strain. *KU70* deficiency did not perturb cell growth although an elevated sensitivity to DNA mutagenic agents was observed. Compared to the other well-known oleaginous yeast, *Yarrowia lipolytica, R. toruloides KU70/KU80* genes contain much higher density of introns and are the most GC-rich *KU70/KU80* genes reported.

**Conclusions:**

The *KU70*-deficient mutant generated herein was effective in improving gene deletion frequency and allowed shorter homology sequences to be used for gene targeting. It retained the key oleaginous and fast growing features of *R. toruloides*. The strain should facilitate both fundamental and applied studies in this important yeast, with the approaches taken here likely to be applicable in other species in subphylum *Pucciniomycotina.*

## Background

*Rhodosporidium toruloides* is a β-carotenoid accumulating oleaginous yeast in subphylum *Pucciniomycotina*[1]. Able to accumulate more than 70% of its dry cell mass as triacylgleride with similar chemical composition to those of plants from ultra-high density fermentation [2-4], *R. toruloides* is regarded as a great host with vast biotechnological potential to produce single cell oil, which may find wide spread applications in staple food, animal feed, biodiesel, surfactant and raw material for industrial polymers [3,5]. Although studies have been done to optimize lipid yield through high-density fermentation [2], there are scarce reports on the rational genetic engineering to improve lipid accumulation or fatty acid profiles in *R. toruloides*. To date, there are no reverse genetic studies reported in *R. toruloides*. With the advent of efficient and stable transformation method established using *Agrobacterium tumefaciens*-mediated transformation (ATMT) in *R. toruloides*[6], reverse genetic studies should become a real possibility.

Targeted gene deletion, often referred as targeted gene knockout, is an essential tool for genetic engineering and reverse genetics. This is an important cornerstone to make any strains commercially competitive [7]. While targeted gene integration in model microorganisms, such as *Saccharomyces cerevisiae* and *Schizosaccharomyces pombe,* can be done with ease and high efficiency [8,9], it is a major obstacle in many industrially important species such as *R. toruloides*.

It has been proposed that DNA repair of double-stranded breaks by homologous recombination (HR) and non-homologous end-joining (NHEJ) operate competitively [10], and the predominance of NHEJ over HR has been regarded as the main cause of low gene targeting efficiency in fungi [11,12]. Correspondingly, one strategy to deal with low gene targeting efficiency in fungi is to improve the HR pathway [11,13]. The other strategy is to inhibit or eliminate the NHEJ pathway, thereby forcing the transformed DNA to be integrated via HR. With this approach, the frequency of HR has been found to be significantly improved with many reports of success in recent years through the disruption of NHEJ pathway by deleting one or more of its key components [12]. In eukaryotes, the main component of the NHEJ system is the DNA-dependent protein kinase (DNA-PK), a three-protein complex consisting of the DNA-dependent protein kinase catalytic subunit (DNA-PKcs) and the regulatory DNA-binding subunits, the Ku70/80 heterodimer [14]. The Ku heterodimer is an abundant nonspecific DNA-binding protein comprising of two tightly-associated subunits of about 70 and 83 kDa, named Ku70 and Ku80 respectively [15]. Both proteins exist in organisms ranging from fungi to human, and are arguably the defining proteins of NHEJ because of their sequence conservation [16].

Here, we report the isolation and characterization of *KU70* and *KU80* homologs in *R. toruloides* and the evaluation of a *KU70*-deficient mutant strain generated for improving gene deletion efficiency in *R. toruloides.*

## Results

### Isolation and characterization of Ku70 and Ku80 encoding genes in *R. toruloides*

**Figure 1:**
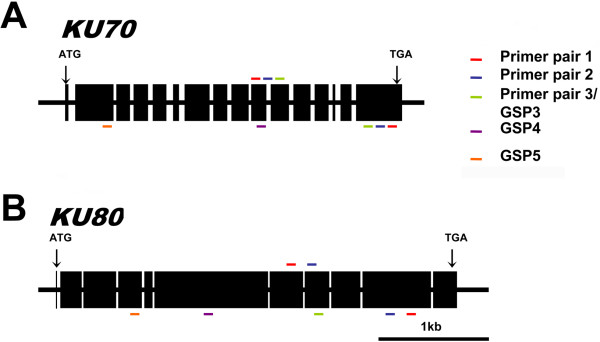
**Genomic organization of *KU70*/*80 *from *R. toruloides*. (A)** Genomic organization of *KU70*. **(B)** Genomic organization of *KU80*. Exons (indicated by black boxes) were identified by comparing the cDNAs and their corresponding genomic DNA sequences. The positions of translation initiation (ATG) and termination (TGA) codons are indicated by arrows. Colored bars indicate positions of gene-specific primers (GSPs) designed for RACEs, with those for 3′ RACE shown on top and those for 5′ RACE shown at the bottom.

Putative genes encoding the Ku70 and Ku80 homologues in the *Rhodotorula glutinis* ATCC 204091 (now re-named as *Rhodosporidium toruloides* ATCC 204091) genome were identified by tBLASTn search against the *R. glutinis* ATCC 204091 genome database at NCBI using the *Ustilago maydis* Ku70 and Ku80 sequences as the query (GenBank acc. no. XP_761295 and XP_761903 respectively). 5′ and 3′ RACEs were performed to obtain the full-length cDNA sequences. The *KU70* cDNA contains a 2,118-nt open reading frame (ORF) flanked by 57-nt and 99-nt 5′ and 3′ untranslated region (UTR) respectively, while the *KU80* cDNA contains a 2,766-nt ORF with 76-nt 5′ UTR and 83-nt 3′ UTR. Comparison of the cDNAs with the genomic sequences revealed that the *KU70* mRNA spans over 3,047 bp containing 16 exons separated by 15 introns, whereas the *KU80* mRNA spans over 3,426 bp containing 11 exons separated by 10 introns (Figure 1). All intronic sequences conformed strictly to the GT-AG rule [17], with a GC content of approximately 61%, which is not significantly different to that of exonic sequences (Table 1). Sequencing of the 3,047 bp *KU70* genomic region in *R. toruloides* ATCC 10657 revealed 100% identity to that of *R. toruloides* ATCC 204091. A comparison with a number of other fungal homologues are shown in Table 1, which shows that *R. toruloides KU70* and *KU80* genes have the highest GC content and highest density of introns (1 in 196 nt on average).

**Table 1:** **Comparison of *KU70*/*80 *** **organization between fungal homologues**

**Gene**	**Strain**	**GenBank accession no.**	**CDS (nt)**	**CDS CG (%)**	**Intron no.**	**Intron CG (%)**	**Average intron length (nt)**	**Reference**
***KU70***	*N. crassa*	AB177394	2046	51.4	2	45.4	54	[18]
	*A. niger*	EF061656	2283	50.7	5	45.4	67	[19]
	*C. neoformans*	XM_573016	1683	48.0	10	46.6	117	[20]
	*Y. lipolytica*	CR382129	1758	48.9	0	-	-	[21,22]
		XM_501610						
	*R. toruloides*	KF850470	2121	59.8	15	61.1	61	This study
***KU80***	*N. crassa*	AB177395	2764	51.2	7	48.3	111	[18]
	*C. neoformans*	XM_568810	2511	47.9	13	43.4	53	[20]
	*Y. lipolytica*	CR382131	2181	48.6	1	37.5	48	[21,22]
		XM_503443						
	*R. toruloides*	KF850471	2769	62.1	10	61.1	66	This study

Note: CDS: coding sequence; nt: nucleotide.

**Figure 2:**
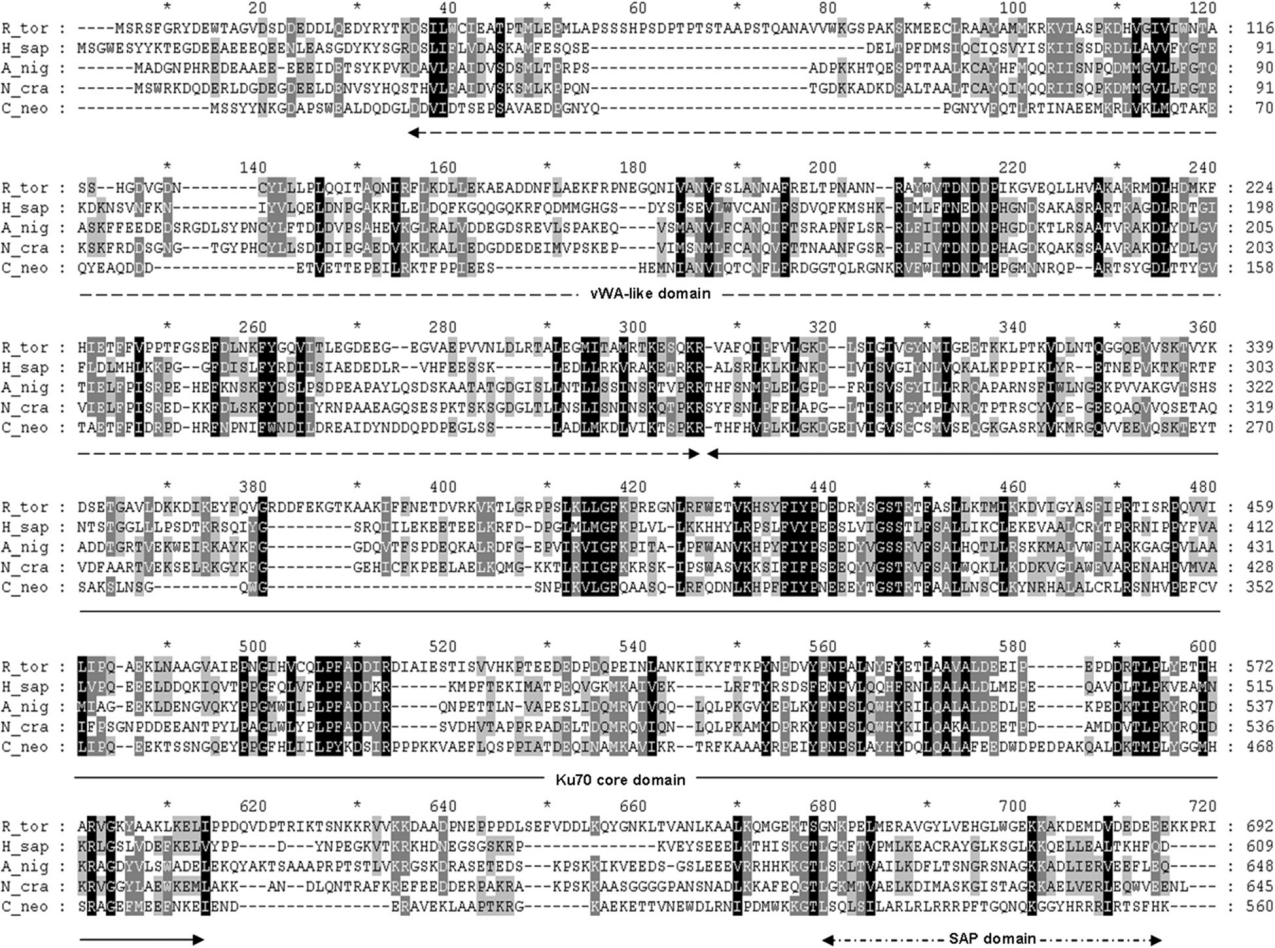
**Sequence comparison of Ku70s.** Multiple sequence alignment of *R. toruloides* Ku70 amino acid sequence (R_tor) with homologues from *Homo sapiens* (H_sap, P12956), *A. niger* (A_nig, ABN13872), *N. crassa* (N_cra, BAD16622) and *C. neoformans* (C_neo, XP_573016). The N-terminal von Williebrand A (vWA)-like domain, a central core domain and the C-terminal SAP (SAF-A/B, Acinus and PIAS) domains are marked with arrow-lines.

The Ku70 ORF sequence was predicted to encode for a protein of 706 amino acids with a molecular weight of 79.5 kDa. Ku70 showed 25% to 30% identities to those from *Homo sapiens*, *Neurospora crassa*, *Aspergillus niger* and *Cryptococcus neoformans*, with the *N. crassa* Ku70 being the closest homologue (Figure 2). Analysis of Ku70 against the SUPERFAMILY database [23] revealed a Ku70 core domain (aa 288–589) that is flanked by a N-terminal “von Willebrand” A (vWA)-like domain (aa 31–54, 82–258), and a C-terminal SAP domain (aa 631–663). The high sequence similarity and presence of signature domains conserved among Ku70 homologues suggest that the characterized Ku70 would be the key component of the NHEJ pathway in *R. toruloides*.

### Targeted gene deletion in wild type *R. toruloides* and generation of *KU70* null mutants

**Figure 3:**
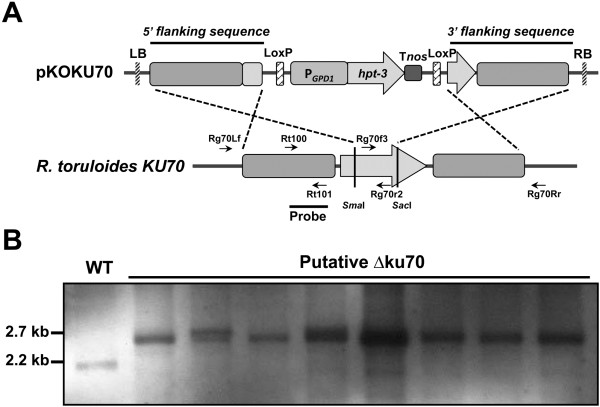
***KU70 *deletion strategy and Southern blot results. (A)** Schematic illustration of *KU70* deletion strategy. LB and RB are the left border and right border sequences of T-DNA derived from pPZP200, respectively; P_*GPD1*_: *R. toruloides GPD1* promoter; *hpt-3*: codon-optimized hygromycin phosphotransferase gene; T_*nos*_: transcriptional terminator of *A. tumefaciens* nopaline synthase gene; *LoxP*: recognition sequences of Cre recombinase; Rg70Lf and Rg70Rr: primers to amplify *KU70* gene deletion region; Rg70f3 and Rg70r2: primers for fungi colony PCR; Rt100 and Rt101: primers to amplify probe used for Southern blot analysis. Unique restriction enzyme digest sites used are shown. **(B)** Southern blot results of putative ∆ku70 transformants. Genomic DNA was digested with *Pvu*I and a band shift from 2.2 kb (WT) to 2.7 kb indicates successful deletion of *KU70*.

To see whether targeted gene deletion could be achieved in wild type *R. toruloides*, *KU70* was used as the first deletion target. A derivative of *R. toruloides* ATCC 10657 (Rt1CE6, named WT hereafter, our unpublished data), which contained a 17β-estradiol inducible *Cre* recombinase gene stably integrated into the genome and allowed the recycling of hygromycin selection marker, was used in ATMT using the *KU70* deletion construct, pKOKU70 (Figure 3A). Eight candidates out of 96 transformants were screened for loss of the targeted deletion region as judged by multiplex PCR (absence of *KU70* PCR product and presence of *GPD1* reference PCR product, data not shown). Further investigation using Southern blot analysis demonstrated that 5 out of 8 candidates were true *KU70* deletion mutants without ectopic integration (Figure 3B). The mutant in lane 2 was therefore named Δku70.

### Gene deletion frequency was improved in the ∆ku70 mutant

While the deletion of *KU70* was obtained with a relatively high frequency (5.2%), deletion of the mating-type specific gene *STE20* and orotidine 5-phosphate decarboxylase gene *URA3*[24,25] proved to be very difficult (Table 2). The low deletion frequency of *STE20* and *URA3* highlighted a need for an improved gene deletion system. To investigate if the Δku70 strain generated earlier could be utilized for this purpose, the hygromycin selection cassette (P_*GPD1*_::*hpt-3*::T_*nos*_) was excised to generate a marker-free *R. toruloides KU70*-deficient derivative (∆ku70e) by activating the Cre recombinase using human hormone 17β-estradiol (Liu et al., unpublished data). As we found that high percentage of 5-fluoroorotic acid (5-FOA) resistant transformants were not true deletion mutants of *URA3* previouly, we decided to evaluate the deletion of *CAR2* homologue as a fast assay for gene deletion frequency because it encodes a bifunctional protein catalyzing phytoene synthase and carotene cyclase that is essential in the biosynthesis of β-carotene [25,26].

**Table 2:** Gene deletion frequency in WT and ∆ku70e strains

**Gene target**	**Homolgy length**^**a**^**(bp)**	**Gene deletion frequency**^**b**^	
		**WT**	**∆ku70e**
*STE20*	800	0 (560)	2.1% (48)
*URA3*	1000	0 (48)	95.8% (48)
*CAR2*	750	10.5% (6152)	75.3% (885)

Note: ^a^Homology sequence length on each side of the hygromycin selection cassette; ^b^Number in parenthesis indicate number of transformants screened.

**Figure 4:**
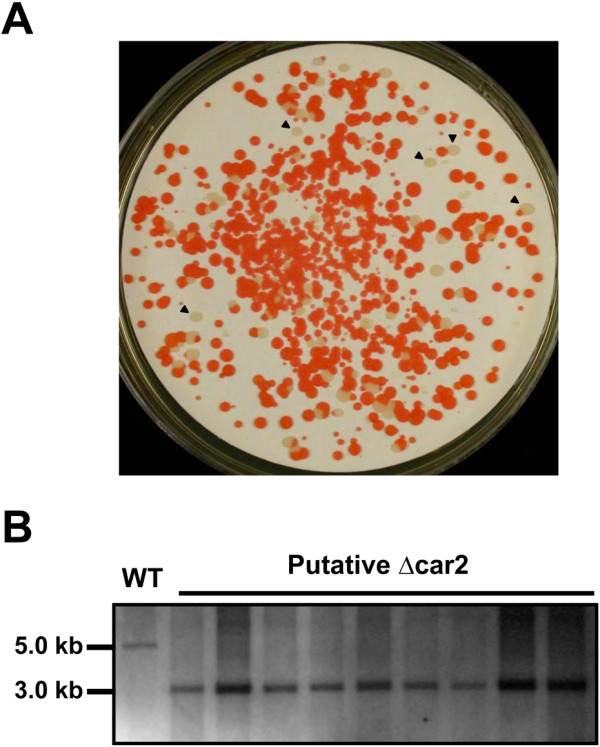
**Phenotypic and genotypic characterization of pKOCAR2 transformants. (A)** A transformation plate showing both red and albino transformants, with black arrow heads marking some albino transformants. **(B)** Southern blot results using the 5′ flanking sequence of *CAR2* as a probe. Genomic DNA was digested with *Pvu*I and a band shift from 5.0 kb (WT) to 3.0 kb indicates successful deletion of *CAR2* gene.

**Figure 5:**
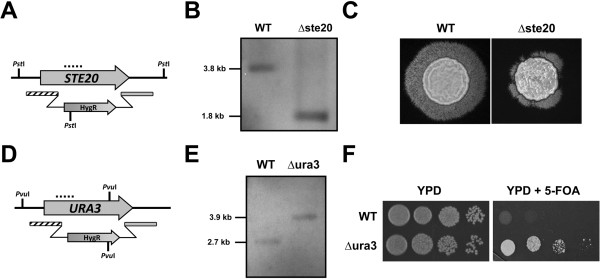
**Targeted deletion of *STE20 *and *URA3 *genes. (A)** and **(D)** Illustration of gene deletion constructs; **(B)** and **(E)**: Southern blots using probes shown in **(A)** and **(D)**; **(C)** Colony phenotype of WT and ∆ste20 strains mated with *R. toruloides* ATCC 10788; **(F)** Growth phenotype of WT and ∆ura3 strains derived from 10-fold serially diluted cells. The latter showed resistance to 5-FOA (1 g/L) – a substrate that can be converted to a toxic intermediate by the *URA3*-encoded enzyme [27].

Using *U. maydis* Car2 [26] as a query for tBLASTn search against the *R. toruloides* ATCC 204091 genome database, a DNA fragment sharing high sequence homology to the query (GenBank acc. no. AVER02000018 from 396838 to 399094-nt, E-value = 1E-23) was identified. *CAR2* was successfully amplified using DNA template of *R. toruloides* ATCC 10657 using oligos Rt079 and Rt080. As expected, albino transformants was observed when WT was transformed with the *CAR2* knockout construct pKOCAR2 (Figure 4A), and the color phenotype of transformants were stable after several rounds of subcultures (data not shown). Multiplex PCR and Southern blot analysis further confirmed that all albino transformants tested were true car2 null mutants (Figure 4B). The albino phenotype was directly caused by the deletion of *CAR2* because the phenotype was completely restored when re-integrating a wild type gene fragment (Additional file 1). Whereas the targeted deletion frequency for *CAR2* was estimated to be 10.5% in WT, it was increased to 75.3% in the ∆ku70e background, a more than 7-fold improvement. Dramatically increased gene deletion frequencies were also observed at both *STE20* and *URA3* loci (Table 2), with the deletions verified by Southern blot and phenotypic analyses (Figure 5).

### Effect of homology sequence length on deletion frequency

To understand the effects of homology sequence length on gene deletion frequency, pKOCAR2 was modified to have various lengths of homology sequence, ranging from 50 to 1500 bp (Additional file 2). The minimum homology length necessary for *CAR2* deletion in WT was at least 250 bp with a gene deletion frequency of 0.7%, while only 100 bp was sufficient in the ∆ku70e strain, which gave gene deletion frequency of approximately 20%. Homology length of at least 1 kb was required to achieve gene deletion frequency of more than 90% using the ∆ku70e strain (Table 3).

**Table 3:** **Effects of homologous sequence length on *CAR2 *** **deletion frequency**

**Homology length (bp)**	**Gene deletion frequency**^**a**^		**Improvement (folds)**
	**WT**	**∆ku70e**	
**50**	0 (780)	0 (8)	-
**100**	0 (620)	21.4% (14)	-
**250**	0.7% (1668)	30.3% (33)	43.3
**500**	11.2% (2124)	67.0% (778)	6
**750**	10.5% (6152)	75.3% (885)	7.2
**1000**	30.4% (2280)	91.7% (2196)	3
**1500**	20.5% (2730)	91.0% (4304)	4.4

Note: ^a^Number in parenthesis indicate number of transformants screened.

### Sensitivity of *KU70* deficient mutant to DNA damaging agents

**Figure 6:**
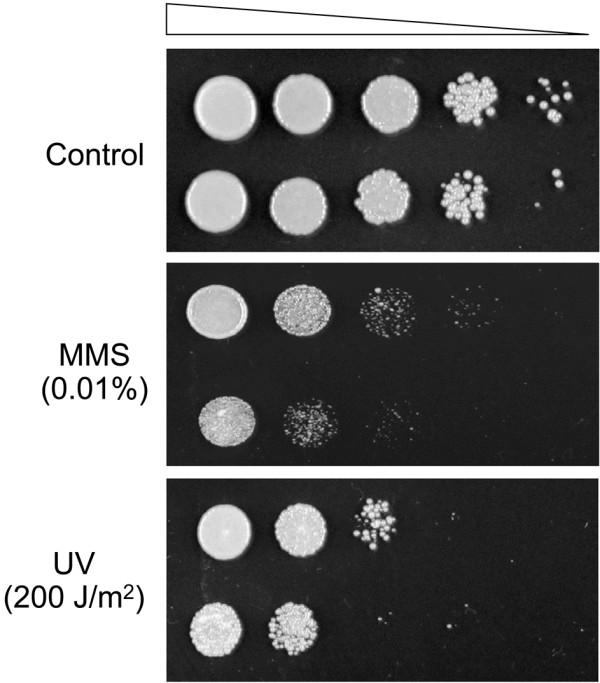
**Sensitivity of WT (top) and *KU70*-deficient strain (bottom) to DNA damaging agents.** An initial cell suspension of OD600 = 1.0 was serially diluted 10 folds for four times and spotted on YPD agar plates containing 0.01% MMS (v/v, upper panel) or subjected to 200 J/m^2^UV irradiation (bottom panel). Top panel shows the non-treated control. All plates were incubated at 28°C for 3 days.

Deficiency in Ku complex encoding genes have been linked to elevated sensitivity to DNA-damaging agents due to the defects in DNA repair [12]. As expected, the ∆ku70 strain displayed higher susceptibility to DNA damage induced by methyl methane sulfonate (MMS) and exposure to ultraviolet (UV) radiation compared to WT. The growth of both strains was repressed when MMS concentration and UV radiation reached 0.01% and 200 J/m^2^ respectively (Figure 6). However, the *KU70*-deficient strain showed no obvious growth defects under normal growth conditions and its cell morphology was indistinguishable from WT. In addition, there were no significant differences in sugar consumption rate and fatty acid profile between WT and ∆ku70 (Additional file 3).

## Discussion

With more than 60% GC content, the *KU70* and *KU80* characterized here present the most GC-rich genes in the NHEJ-pathway reported so far. In terms of gene structure, both genes contain much higher density of introns than those of *Y. lipolytica* (Table 1), which is the best-studied oleaginous yeast to date. Not surprisingly, homologues of *C. neoformans,* which is under the same *Basidiomycota* phylum, also have high density of introns (Table 1).

DSB repair can differ in heterochromatic and euchromatic regions of the genome and histone modifying factors play an important role in this process [28,29]. Recombination frequencies are known to vary in different genes even when assayed with the same technique and in the same genetic background [30]. Impairment of the NHEJ-pathway has proved to be effective in improving homologous recombination frequency in many eukaryotic hosts. However, the magnitude of improvement appears to vary considerably in different reports. With a homology sequence of approximately 750 bp, the *CAR2* deletion frequency was improved 7.2-fold, from 10.5%, in WT to 75.3% in the *KU70*-deficient mutant in *R. toruloides*. This is similar to the deletion of *TRP1* in *Y. lipolytica* although substantially higher knockout frequencies have been reported for several genes in other fungi, for example, *N. crassa, A. niger and C. neoformans* (Additional file 4). Nevertheless, the *R. toruloides STE20* gene remained very difficult to knockout even with the ∆ku70e mutant (Table 2). This demonstrates a positional effect and implies additional factors that regulate gene deletion in *R. toruloides.* As the *STE20* gene is located between the mating type loci *RHA2* and *RHA3* in *R. toruloides*[24], it is possible that the gene is within a transcriptionally silenced chromatin as was reported for the mating type genes in a number of other fungi [31,32]. The low deletion frequency of *STE20* suggests a potential role of chromatin structure and/or gene expression level in regulating DNA recombination in *R. toruloides.*

One of the drawbacks of NHEJ-deficient strains is its elevated sensitivity to DNA damage and the possibility of generating unwanted mutations [12]. Indeed, the *KU70*-deficient strain studied here showed increased sensitivity to MMS and UV radiation. However, the mutant did not show severe growth defects under normal growth conditions. With comparable sugar consumption rate and fatty acid profile to the WT, the ∆ku70 and ∆ku70e strains should maintain much of the appeal of *R. toruloides* in industrial applications.

## Conclusions

The *KU70*-deficient mutant generated herein was found to be effective in improving gene deletion frequency and retained the key oleaginous and fast growing features of *R. toruloides*. The strain should facilitate both fundamental and applied studies in this important yeast, with the approaches taken here likely to be applicable in other species in subphylum *Pucciniomycotina.*

## Methods

### Strains, media, and culture conditions

*R. toruloides* strain ATCC 10657 and ATCC 204091 (previously named *Rhodotorula glutinis*) were purchased from American Type Culture Collection (ATCC, Manassas, VA, USA) and cultured at 28°C in YPD broth (1% yeast extract, 2% peptone, 2% glucose, w/v) or on potato-dextrose agar (PDA). *A. tumefaciens* strain AGL1 [33] was grown at 28°C in either liquid or solid 2YT medium (1.6% tryptone, 1% yeast extract, 0.5% NaCl, pH 7.5). *Escherichia coli* XL1-Blue was cultured at 37°C in Luria-Bertani (LB) broth or on LB agar for routine recombinant DNA work.

### Rapid amplification of cDNA ends (RACE)

The SMARTer™ RACE cDNA Amplification Kit (Clontech, Mountain CA, USA) was used to determine the full-length sequences of *KU70* and *KU80* RNA transcripts according to the manufacturer’s instruction. For *KU70*, oligonucleotides Rg70r3 and Rg70f3 were used as gene-specific primers for 5′ and 3′ RACE respectively. Two more steps of 5′ RACE using oligos Rg70r4 and Rg70r5 were performed before the full-length cDNA sequence was assembled. Similarly, oligos Rg80r2 and Rg80f2 were used as gene specific primers for 5′ and 3′ RACE for *KU80* respectively. Another two steps of 5′ RACE were performed using primers Rg80r3 and Rg80r4 to assemble the complete cDNA sequence. All oligonucleotides used are listed in Table 4.

**Table 4:** Oligonucleotides used

**Name**	**Sequence (5′ to 3′)**	**Specificity**
Rg70f3	GTTCATCCCGAGGACCATCAGTC	Sense primer for *KU70* 3′ RACE Specific primer for ∆ku70 fungi colony PCR
Rg70r3	GGGATGAGCTCCTTGAGCTTTGC	Antisense primer 1 for *KU70* 5′ RACE
Rg70r4	TCTTCGTCCGGGTAGATGAAGTAG	Antisense primer 2 for *KU70* 5′ RACE
Rg70r5	ATCCTTTGGGCTGGCGATGACTTTG	Antisense primer 3 for *KU70* 5′ RACE
Rg80f2	GTCGCTTGAGCGCATCTTCAACAAG	Sense primer for *KU80* 3′ RACE
Rg80r2	ATCACGATGTCCTCGTCCTCGTCGT	Antisense primer 1 for *KU80* 5′ RACE
Rg80r3	CTCCAACCTGCGGACATGCTTCTT	Antisense primer 2 for *KU80* 5′ RACE
Rg80r4	GAAGCGAGCCTTGCGCTTGATCTCG	Antisense primer 3 for *KU80* 5′ RACE
Rg70Lf	CTCGTGTCAAGAAAACCAGCAAG	*KU70* gene deletion region
Rg70Rr	ACCTCAGCAACCAACCTACCAGT	
C50f	TCCTCCTACCGACGCTCTACC	*CAR2* deletion region (50 bp homology length)
C50r	CTCTGTGCAGCCATCTCGTTC	
C100f	TTCATTGCCTCAACCCACATC	*CAR2* deletion region (100 bp homology length)
C100r	CGTCCCCAATGCTGAGATAAA	
C250f	CAAAGTTGGAGGAGGGAGGAG	*CAR2* deletion region (250 bp homology length)
C250r	GTTGAGAAACCAGCGAGACCA	
C500f	CCTGGGACTCGTACCTCATCC	*CAR2* deletion region (500 bp homology length)
C500r	GCGACCGCGACTGACTAGTTT	
Rt079	GGACTGGACTACTGGCTCGTGT	*CAR2* deletion region (750 bp homology length)
Rt080	TCAAGAGCTACCAGGAGAGCAAC	*CAR2* deletion region (750 bp homology length) *CAR2* complementation region
C1000f	GGGGCTGTCTGTGACTTGCTAT	*CAR2* deletion region (1000 bp homology length)
C1000r	TCGAGCTCAAGCTGAAGAAGAAG	
C1500f	CTCTCGCACGGGGAGGTAGT	*CAR2* deletion region (1500 bp homology length) *CAR2* complementation region
C1500r	TGCTTGCCAAGAAGCCTACAATA	*CAR2* deletion region (1500 bp homology length)
STE20Lf	GCTCGTGTTGCGGGAACAGT	*STE20* deletion region
STE20Rr	GGGCACGCCTTCTTCAAGAAC	
Rt033	GTCAAGCCGCCCAGGCTGTC	*URA3* deletion region
Rt034	GGATCCGCCAAGTCGCGCAG	
Rg70r2	GTCCTTCTTCACCACGCGTTTCTT	Specific primer for ∆ku70 fungi colony PCR
Rt096	CGAATCCAACATCGACATGATTT	Specific primer for ∆car2 fungi colony PCR
Rt097	TATTGACGAGCTGAAGAGCCTGT	
Rt006	TCTCCCTCGCCCTCTGCT	Specific primer for *GPD1* (reference gene) in multiplex PCR
Rt007	AGCCATGCCGGTGAGCTTG	
Rt100	GACCCGACAGACGAGTGGAC	*KU70* probe for Southern blot
Rt101	AGGTCCTTGAGAAAGCGGATG	
Rt083	CTCACCCTCGTGTTGCTCGTA	*CAR2* probe for Southern blot
Rt084	CCTCTCCTCCCTCCTCCAACT	

### DNA constructs

All restriction and modification enzymes used were from New England Biolabs Inc. (NEB, Ipswich, MA, USA), unless otherwise stated. Plasmid pEX2 is a pPZP200 derivative routinely used as the binary T-DNA vector backbone [34].

pEX2 was digested with *Sac*I (blunt-ended) and *Pme*I to remove the hygromycin resistant gene cassette (P*gpd*::*hpt*::T_*35S*_), and inserted with the 3,618 bp 5′-phosphorylated *KU70* DNA fragment amplified from genomic DNA of *R. toruloides* ATCC 10657 using oligos Rg70Lf and Rg70Rr to create pEX2KU70. pDXP795hptR contained a hygromycin selection cassette composed of the endogenous *GPD1* promoter from *R. toruloides* (795 bp version), codon-optimized hygromycin phosphotransferase gene (JQ806387, *hpt-3*) and the terminator of nopaline synthase gene of *A. tumefaciens* (P_*GPD1*_*::hpt-3::*T_*nos*_, Additional file 5A), and the selection cassette is flanked at both ends by *loxP* sites, allowing its deletion by Cre recombinase that can be activated when required (Liu et al., unpublished data). To create *KU70* deletion vector pKOKU70, hygromycin selection cassette was digested from pDXP795hptR by *Bam*HI-*Hin*dIII and the blunt-ended fragment was inserted into *Sma*I-*Sac*I (blunt-ended) sites of pEX2KU70. Similarly, pKOCAR2 was constructed first by cloning the 2,697 bp 5′-phosphorylated *CAR2* fragment into pEX2, which was amplified using oligos Rt079 and Rt080. Subsequently, the P_*GPD1*_::*hpt-3*::T_*nos*_ cassette was inserted between *Sac*II-*Apa*I (blunt-ended) sites, generating pKOCAR2 containing a homologous flanking sequence of approximately 750 bp at both ends. Gene deletion vectors for *CAR2* carrying homology sequence of various lengths (50, 100, 250, 500, 750, 1000 and 1500 bp) were likewise constructed using oligonucleotides listed in Table 4 (Additional file 2). For *CAR2* complementation, a 3,242 bp fragment amplified by oligos C1500f and Rt080 was 5′-phosphorylated and inserted to *Hin*dIII digested and blunt-ended pDXP795hptR to generate the complementation plasmid (Additional file 5B).

Using the same strategy for gene deletion vectors, the deletion region of *STE20* and *URA3* were amplified using oligos STE20Lf/STE20Rr (2,196 bp) and Rt33/Rt34 (2,784 bp), cloned into pEX2 and digested using *Bsp*HI/*Nco*I and *Stu*I/*Mfe*I (blunt-ended) to create pKOSTE20 and pKOURA3, respectively.

### Transformation and identification of transformants

ATMT and fungal colony PCR were both performed as described previously [6]. For further identification of gene deletion mutants, multiplex PCR [35] using genomic DNA as the template was performed to prevent false negative results. Two sets of primer pairs, one specific to the deletion target (Rg70f3/Rg70r2 and Rt096/Rt097 for *KU70* and *CAR2* gene, respectively) and the other to the reference gene *GPD1* (Rt006 and Rt007) were added to the reactions.

### Isolation of genomic DNA, RNA and Southern blot analysis

Cell cultures at exponential stage were collected and genomic DNA was extracted using MasterPure™ Yeast DNA purification kit (Epicentre, Madison, WI, USA), while RNA was extracted as described previously [6]. The concentrations of extracted DNA or RNA samples were determined with NanoDrop® ND-1000 Spectrophotometer (Thermo Scientific, Wilmington, DE, USA) and their integrity were checked by agarose gel electrophoresis.

For Southern blot analysis, 10 μg of genomic DNA was digested with *Pvu*I at 37°C for about 24 hrs and resolved by electrophoresis in a 0.8% agarose gel. Southern hybridization and detection procedures were performed using DIG (digoxigenin)-High Prime DNA Labeling and Detection kit in accordance with the manufacturer’s instructions (DIG Application Manual for Filter Hybridization, Roche Diagnostics, Indiana, IA, USA). The probes were amplified by PCR labeling using DIG DNA labeling mix, with primers Rt100 and Rt101 used to amplify a fragment targeting the 5′ flanking sequence of *KU70*, and Rt083 and Rt084 specific to the 5′ flanking sequence of *CAR2*.

### Sensitivity to DNA-damaging agents

MMS and UV radiation were the DNA-damaging agents used to analyze strain sensitivity monitored by spot plate assay. Cell cultures in YPD broth were adjusted to one OD_600_ unit and 10-fold serial diluted, from which the diluted samples were spotted on YPD agar plates supplemented with MMS (Sigma, MO, USA) ranging from 0.001-0.1%. Exposure to UV radiation was done by placing the plates in a UV Crosslinker (Spectrolinker™ XL-1000, Spectronics Corporation, NY, USA) at a dose ranging from 100 to 600 J/m^2^ after the samples were spotted.

### Photomicroscopy

Freshly cultured cells were analyzed using a Nikon Eclipse 80i microscope equipped with CFI Plan Apochromat objectives (Nikon, Melville, NY, USA). Images were acquired with a Nikon DS camera interfaced with NIS-Element F 3.0 software.

### GenBank accession numbers

The annotated *KU70* and *KU80* sequences from *R. toruloides* ATCC 204091 have been deposited in GenBank under the accession number of KF850470 and KF850471, respectively.

## Competing interests

The authors declare that they have no competing interests.

## Authors’ contributions

CMJK, LJ, and YL designed the experiments and prepared the manuscript. CMJK, YL, HSM and MD performed the experiments. CMJK, YL and LJ analyzed the data. All authors read and approved the final manuscript.

## Supplementary Material

Additional file 1 **Colony colors of ∆car2e after being transformed with a wild type copy of the *R. toruloides CAR2 *** **genomic DNA fragment.** ∆car2e is a hygromycin sensitive derivative of a *CAR2* targeted deletion mutant made by activating the *Cre* recombinase gene stably integrated into the genome. Click here for file

Additional file 2 **Schematic diagram of *CAR2 *** **deletion constructs with varied homology length sequence ranging from 50 to 1500 bp used to compare the homologous recombination frequencies between WT and *KU70***-**deficient strain.** Restriction enzyme digest sites used for cloning and Southern blot analysis are indicated. The components in the diagram are not drawn to scale. Click here for file

Additional file 3 **Comparisons of WT and ∆ku70 strains. (A)** Cell morphology; **(B)** growth rate; **(C)** sugar consumption rates; **(D)** fatty acid profiles. Click here for file

Additional file 4 **Comparison of gene deletion frequency between different WT and *KU70*-deficient fungal stains.** Click here for file

Additional file 5 **(A) Schematic illustration of T-DNA region of pDXP795hptR.** Unique restriction enzyme digest sites used are shown. **(B)** Schematic illustration of *CAR2* complementation plasmid within T-DNA region. Click here for file
