# Supplementary figures and images for: Molecular characterization of KU70 and KU80 homologues and exploitation of a KU70-deficient mutant for improving gene deletion frequency in Rhodosporidium toruloides
Source: BMC Microbiol. 2014 Feb 27;14:50. doi: 10.1186/1471-2180-14-50 (PMC4101874; doi:10.1186/1471-2180-14-50)

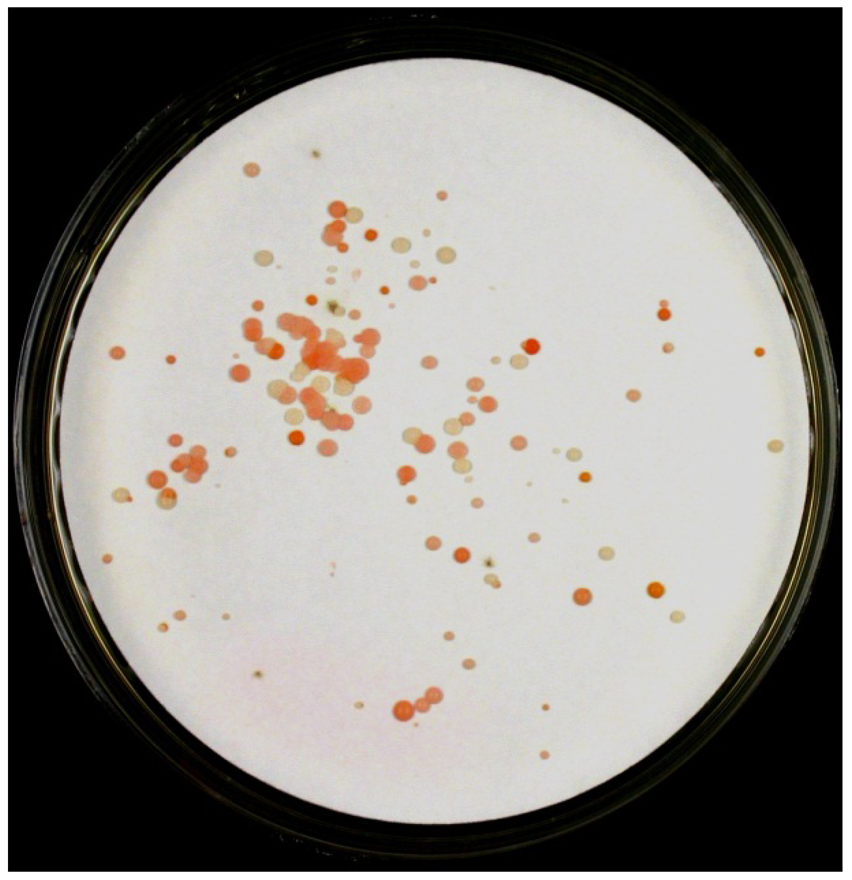

Supplement: Additional file 1 — Colony colors of ∆car2e after being transformed with a wild type copy of the R. toruloides CAR2 genomic DNA fragment. ∆car2e is a hygromycin sensitive derivative of a CAR2 targeted deletion mutant made by activating the Cre recombinase gene stably integrated into the genome. [file 1471-2180-14-50-S1.tiff]

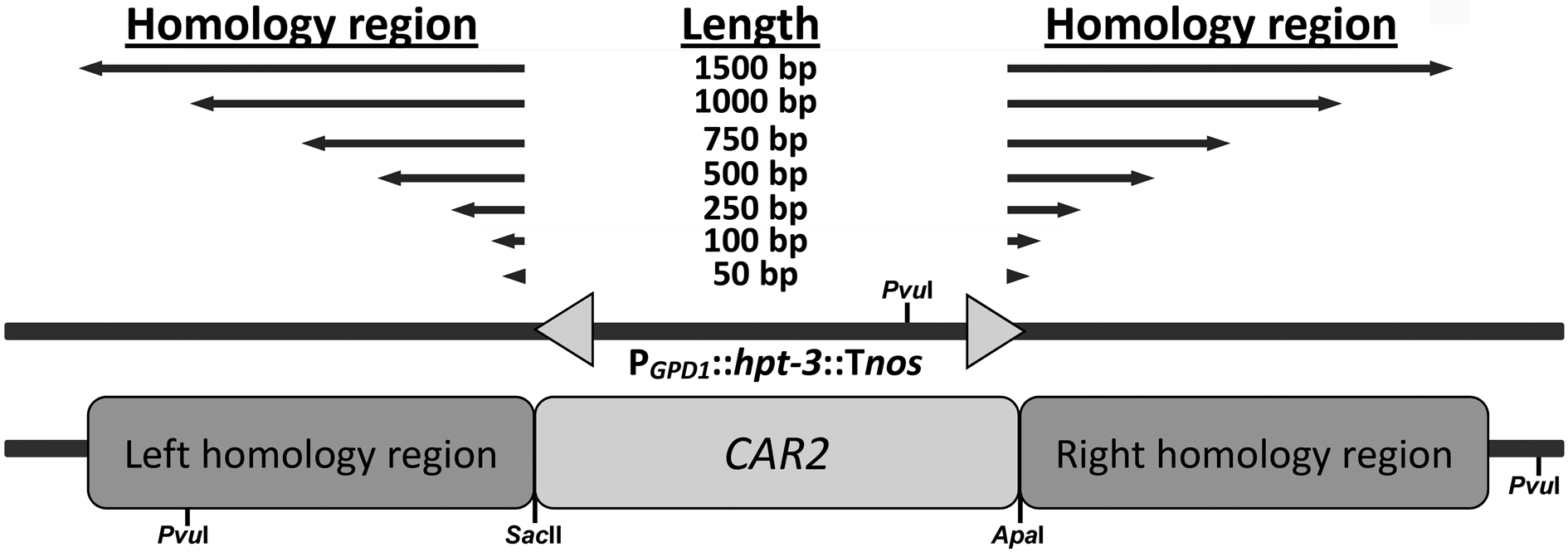

Supplement: Additional file 2 — Schematic diagram of CAR2 deletion constructs with varied homology length sequence ranging from 50 to 1500 bp used to compare the homologous recombination frequencies between WT and KU70-deficient strain. Restriction enzyme digest sites used for cloning and Southern blot analysis are indicated. The components in the diagram are not drawn to scale. [file 1471-2180-14-50-S2.tiff]

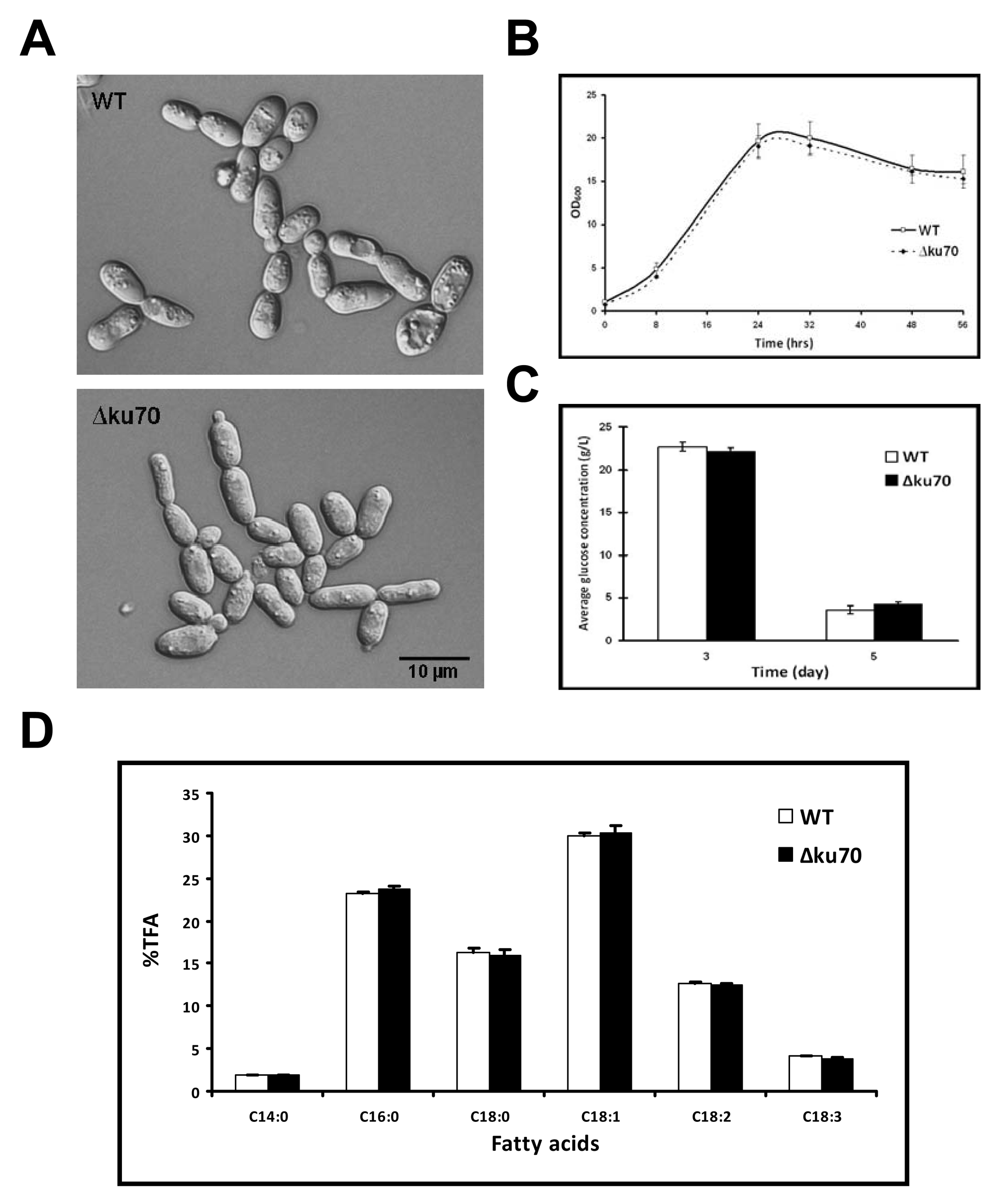

Supplement: Additional file 3 — Comparisons of WT and ∆ku70 strains. (A) Cell morphology; (B) growth rate; (C) sugar consumption rates; (D) fatty acid profiles. [file 1471-2180-14-50-S3.tiff]

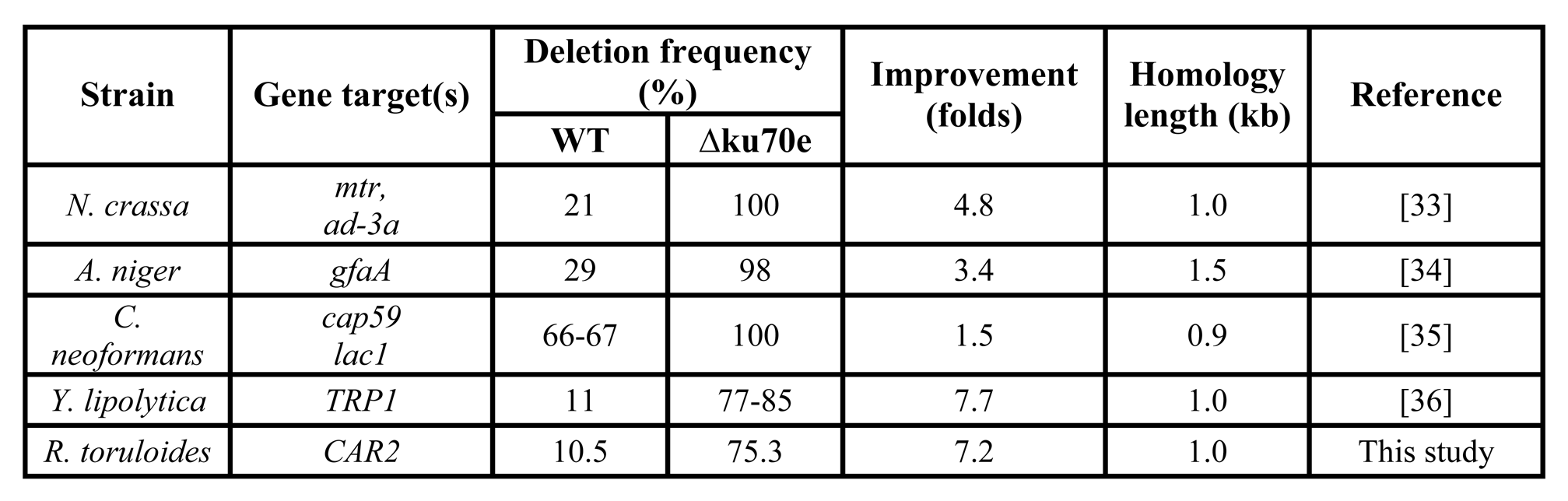

Supplement: Additional file 4 — Comparison of gene deletion frequency between different WT and KU70-deficient fungal stains. [file 1471-2180-14-50-S4.tiff]

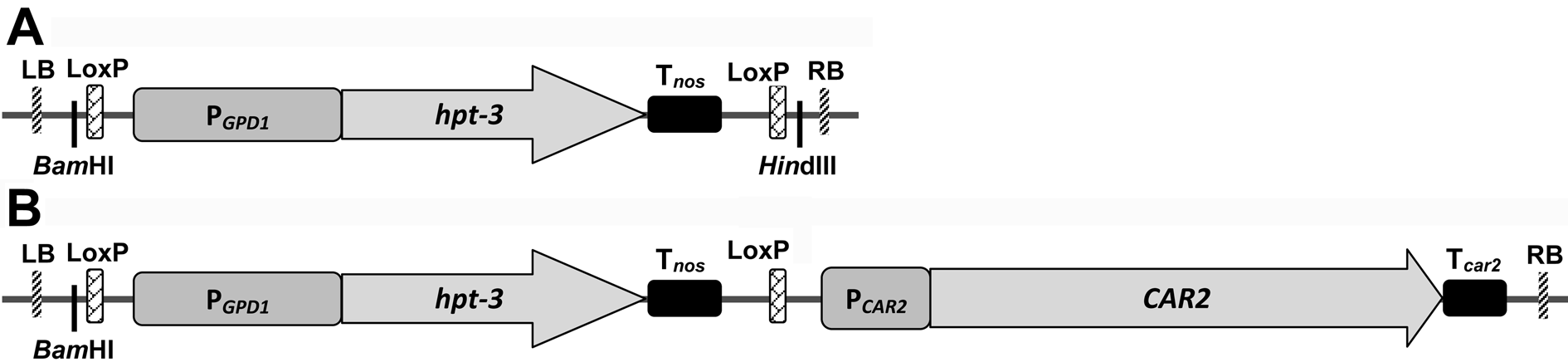

Supplement: Additional file 5 — (A) Schematic illustration of T-DNA region of pDXP795hptR. Unique restriction enzyme digest sites used are shown. (B) Schematic illustration of CAR2 complementation plasmid within T-DNA region. [file 1471-2180-14-50-S5.tiff]
